# Supplementary material for: Terpenoids of the Swamp Cypress Subfamily (Taxodioideae), Cupressaceae, an Overview by GC-MS
Source: Molecules. 2019 Aug 21;24(17):3036. doi: 10.3390/molecules24173036 (PMC6751496; doi:10.3390/molecules24173036)
Supplement: Supplementary file 1 [file molecules-24-03036-s001.pdf]

Supplemental Material

for

Terpenoids of the swamp cypress subfamily (Taxodioideae), Cupressaceae, an overview by GC-MS

Bernd R. T. Simoneit[1]*, Angelika Otto[2], Daniel R. Oros[3], and Norihisa Kusumoto[4]

[1] Department of Chemistry, College of Science, Oregon State University, Corvallis, OR 97331, USA

[2] Forschungsinstitut Senckenberg, Sektion Paläobotanik, Senckenberganlage 25, D-60325 Frankfurt am Main, Germany

[3] Consultant, 72 Marina Lakes Drive, Richmond, CA 94804, USA

[4] Wood Extractive Laboratory, Forestry and Forest Products Research Institute, 1 Matsunosato, Tsukuba, Ibaraki 305-8687, Japan

Contents

Figures SM-1 through SM-7. Mass spectra of the terpenoids from the Taxodioideae resins (ordered in ascending molecular weights).

Figure SM-1. Mass spectra of the terpenoids from the Taxodioideae resins.

(a) Sugiol (a4, standard),

(b) 11-Hydroxy-12-oxoabieta-7,9(11),13-triene (a.k.a. 6-deoxotaxodione, a6) or 11-hydroxy-6,7-dehydroferruginol (a6),

(c) 6,7-Dehydroferruginol (a1),

(d) Pisiferol (a7),

(e) *abeo*-Pisiferol (a8),

(f) Taxodione (a10, R=H, standard),

(g) Salvinolone (a11, standard),

(h) Sugiol methyl ether (a4, Me, standard).

Figure SM-2. Mass spectra of the terpenoids from the Taxodioideae resins.

(a) 6,7-Dehydroroyleanone (a12, standard),

(b) Taxodone (a14, standard),

(c) 11-Hydroxysugiol (a15),

(d) Royleanone (a13, Standard),

(e) *abeo*-Carnosol (a16),

(f) 6-Hydroxysalvinolone (14-deoxycoleon U, a17, standard),

(g) Methyl pisiferate,

(h) Methyl imbricataloate (L4, standard).

Figure SM-3. Mass spectra of the terpenoids from the Taxodioideae resins.

(a) Methyl imbricatoloate (L7, standard),

(b) Taxodione acetate (a10, R=Ac),

(c) Dimethyl dihydroagathate (L8, standard),

(d) 7-Acetoxy-6,7-dehydroroyleanone (a25, standard),

(e) Sugiol-TMS (a4, standard),

(f) 11-Hydroxy-12-oxoabieta-7,9(11),13-triene-TMS (a6),

(g) *abeo*-Pisiferol-TMS (a8),

(h) Taxodione-TMS (a10, R=H, standard).

Figure SM-4. Mass spectra of the terpenoids from the Taxodioideae resins.

(a) 6,7-Dehydroroyleanone-TMS (a12, standard),

(b) Royleanone-TMS (a13, standard),

(c) Imbricataloic acid-TMS (L4, standard),

(d) Methyl imbricatoloate-TMS (L7, standard),

(e) 24-Methylenecycloartan-3-one (T1),

(f) 7-Acetoxy-6,7-dehydroroyleanone-TMS (a25, standard),

(g) 11-Hydroxy-6,7-dehydroferruginol-TMS (a6),

(h) Pisiferol-TMS (a7).

Figure SM-5. Mass spectra of the terpenoids from the Taxodioideae resins.

(a) 11-Hydroxyferruginol-TMS (a9),

(b) 7-Acetoxyroyleanone-TMS (a25, standard),

(c) Chamaecydin (a27, standard),

(d) *Iso*-chamaecydin (a28),

(e) 24-Ethylenecycloartan-3-one (T2),

(f) Salvinolone-TMS (a11, standard),

(g) Taxodone-TMS (a14, standard),

(h) 11-Hydroxysugiol-TMS (a15).

Figure SM-6. Mass spectra of the terpenoids from the Taxodioideae resins.

(a) *abeo*-Carnosol-TMS (a16),

(b) 13-*epi*-Cupressic acid-TMS (L5),

(c) *Iso*-cupressic acid-TMS (L6),

(d) 6β-Hydroxychamaecydin (a29, standard),

(e) Imbricatoloic acid-TMS (L7, standard),

(f) Taxoquinone-TMS (a18),

(g) Horminone-TMS (a20),

(h) 13,14-Dihydroagathic acid-TMS (L8, standard).

Figure SM-7. Mass spectra of the terpenoids from the Taxodioideae resins.

(a) Chamaecydin-TMS (a27, standard),

(b) *Iso*-chamaecydin-TMS (a28),

(c) 6-Hydroxysalvinolone-TMS (a17, standard),

(d) 6β,11-Dihydroxysugiol-TMS (a22),

(e) 7-Hydroxytaxodone-TMS (a19),

(f) 6α-Hydroxytaxoquinone-TMS (a23, standard),

(g) 6β-Hydroxytaxoquinone-TMS (a24, standard),

(h) 6β-Hydroxychamaecydin-TMS (a29, standard).

Figure SM-1.

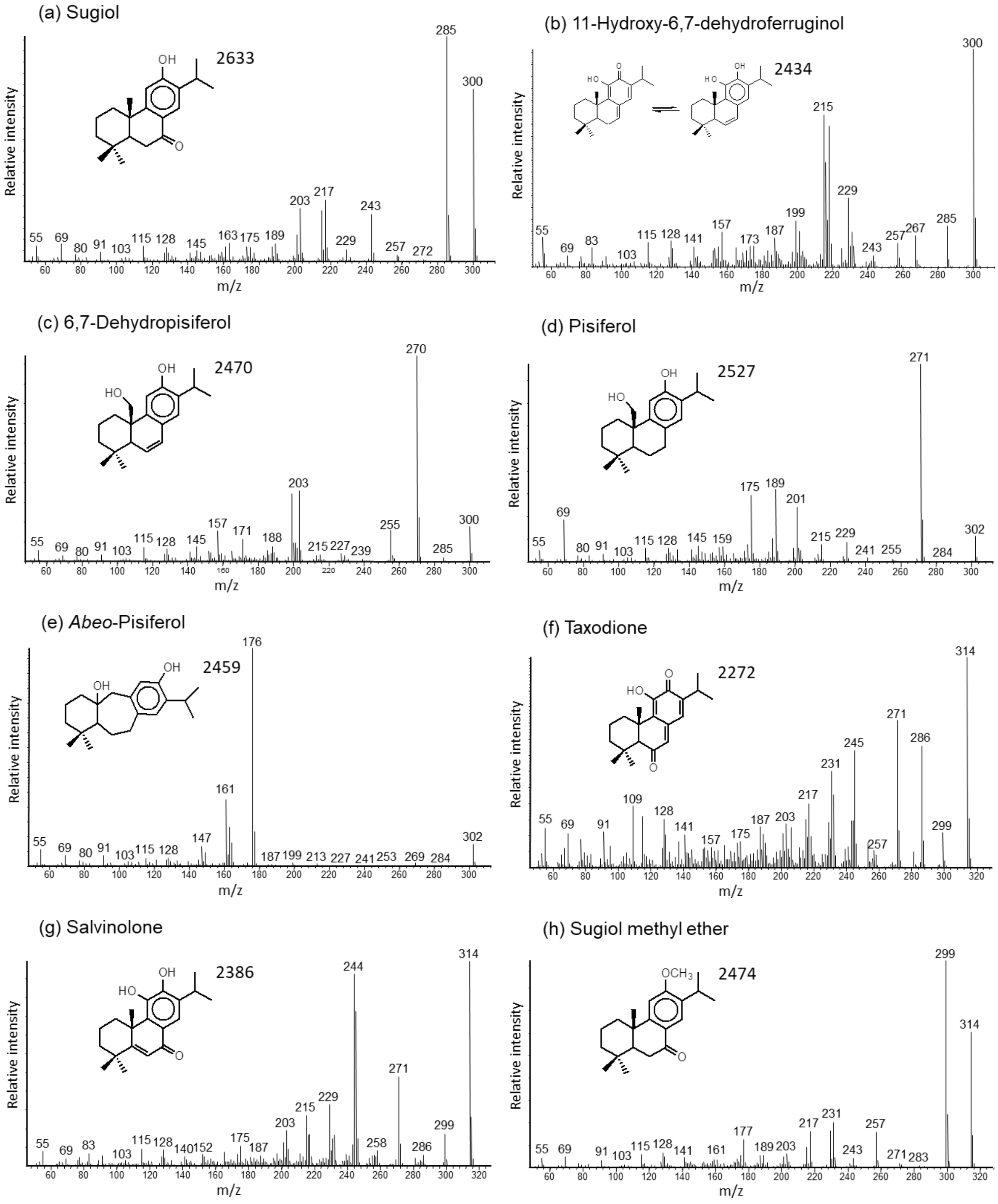
(a) Sugiol
2633
(b) 11-Hydroxy-6,7-dehydroferruginol
2434
(c) 6,7-Dehydropisiferol
2470
(d) Pisiferol
2527
(e) Abeo-Pisiferol
2459
(f) Taxodione
2272
(g) Salvinolone
2386
(h) Sugiol methyl ether
2474
Relative intensity
m/z

Figure SM-2.

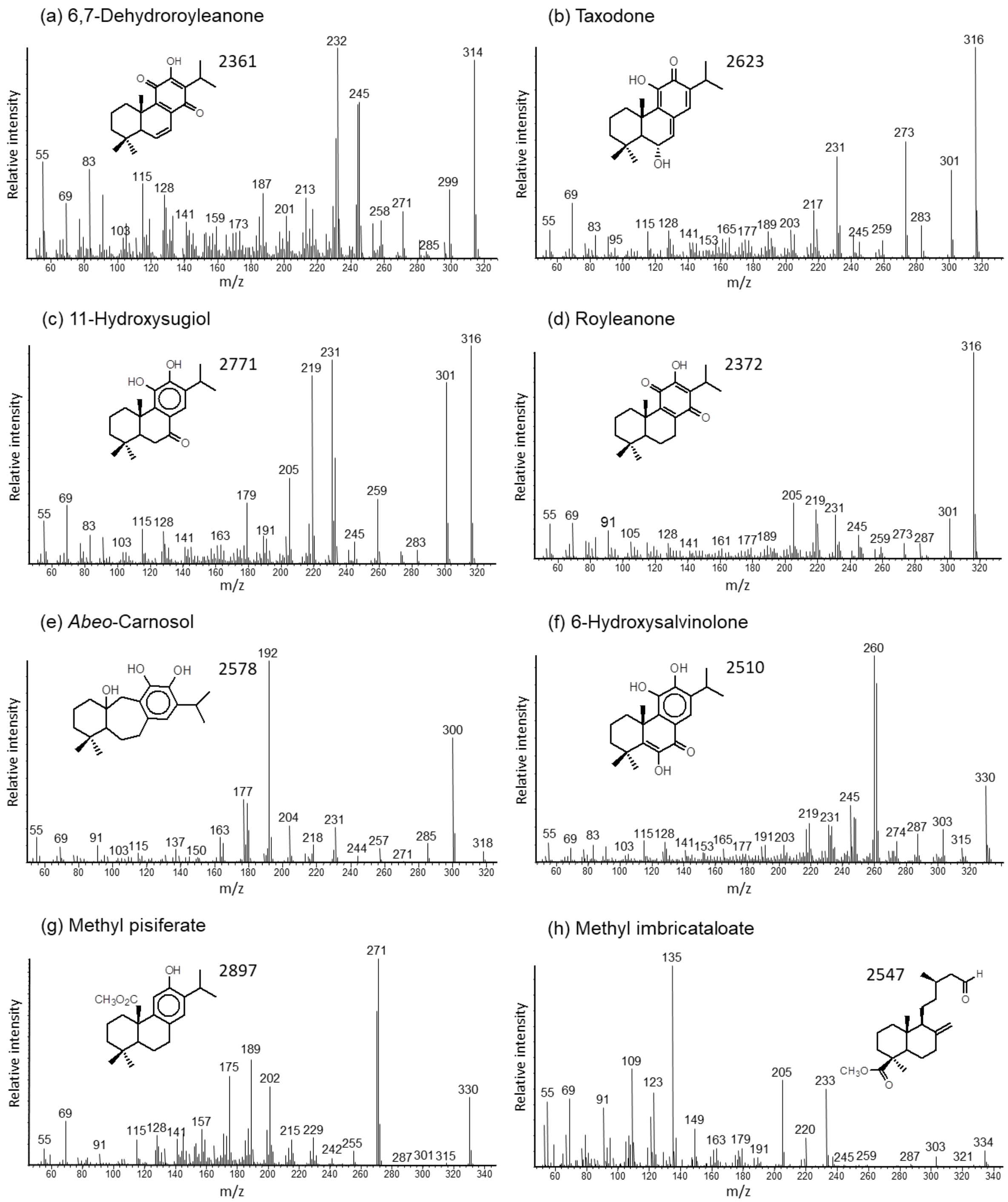
(a) 6,7-Dehydroroyleanone
2361
(b) Taxodone
2623
(c) 11-Hydroxysugiol
2771
(d) Royleanone
2372
(e) *Abeo*-Carnosol
2578
(f) 6-Hydroxysalvinolone
2510
(g) Methyl pisiferate
2897
(h) Methyl imbricataloate
2547
Relative intensity
m/z

Figure SM-3.

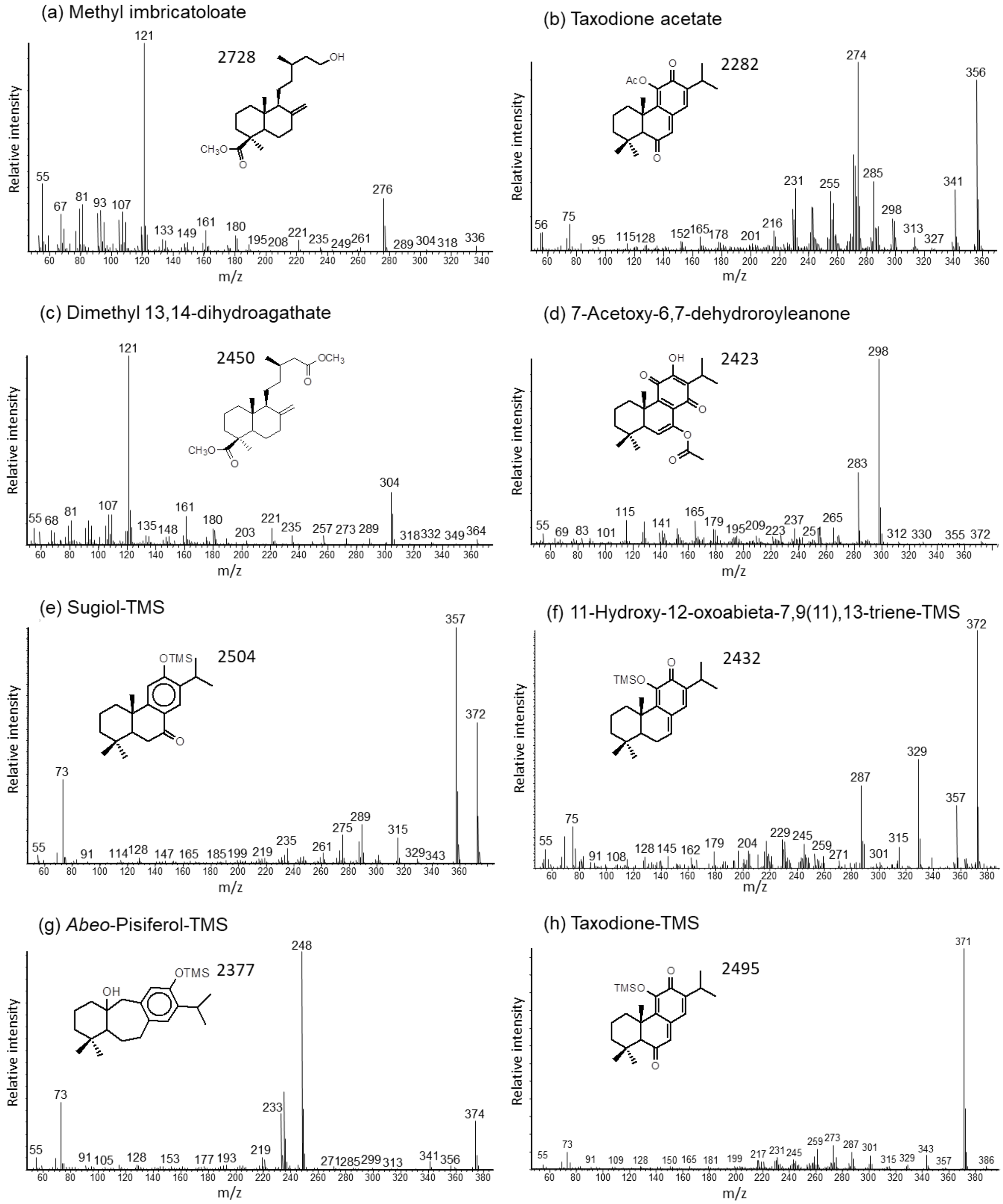
(a) Methyl imbricatoloate
2728
(b) Taxodione acetate
2282
(c) Dimethyl 13,14-dihydroagathate
2450
(d) 7-Acetoxy-6,7-dehydroroyleanone
2423
(e) Sugiol-TMS
2504
(f) 11-Hydroxy-12-oxoabieta-7,9(11),13-triene-TMS
2432
(g) *Abeo*-Pisiferol-TMS
2377
(h) Taxodione-TMS
2495
Relative intensity
m/z

Figure SM-4.

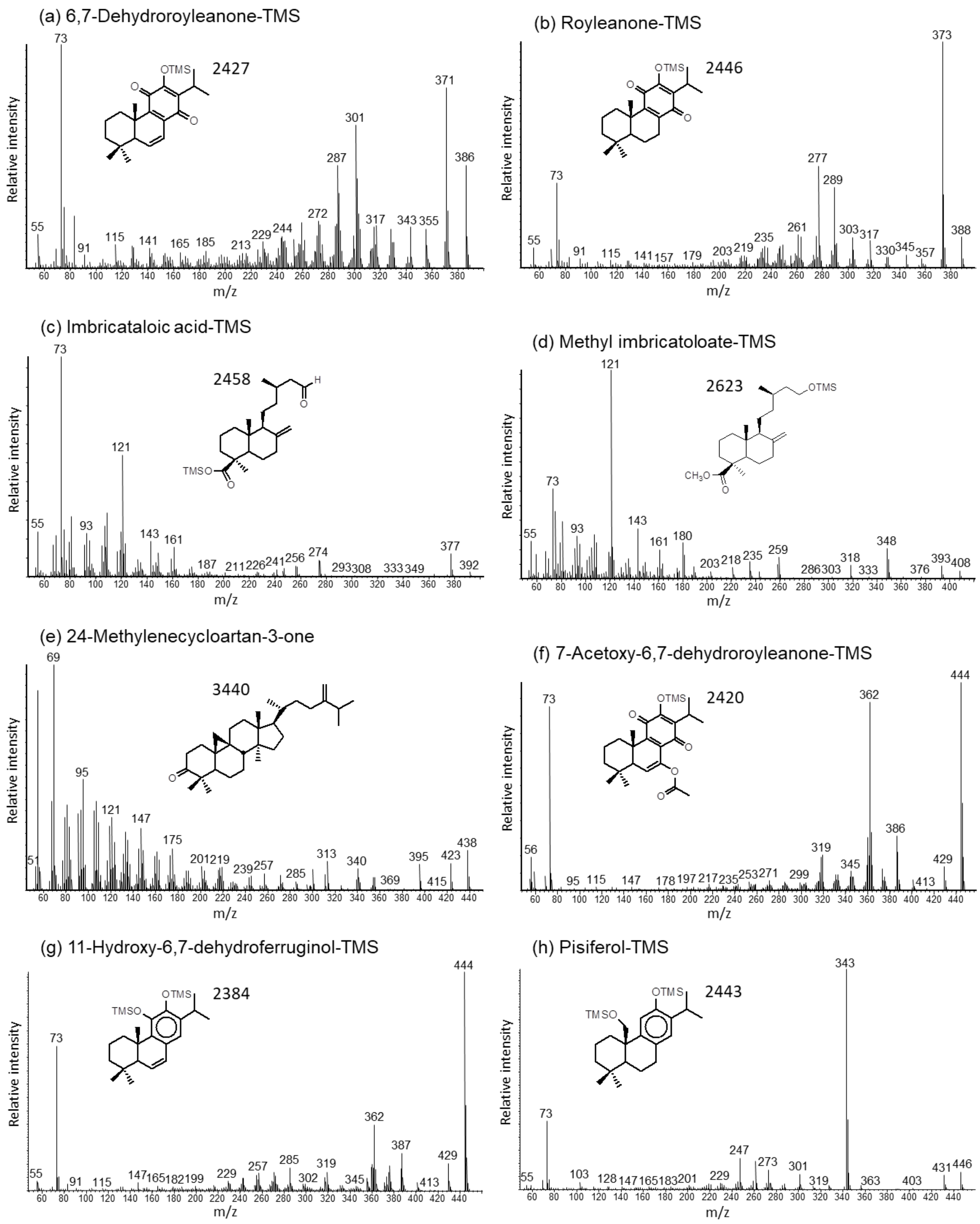
(a) 6,7-Dehydroroyleanone-TMS
2427
OTMS
(b) Royleanone-TMS
2446
OTMS
(c) Imbricataloic acid-TMS
2458
TMSO
(d) Methyl imbricatoloate-TMS
2623
OTMS
CH_3O
(e) 24-Methylenecycloartan-3-one
3440
(f) 7-Acetoxy-6,7-dehydroroyleanone-TMS
2420
OTMS
(g) 11-Hydroxy-6,7-dehydroferruginol-TMS
2384
TMSO
OTMS
(h) Pisiferol-TMS
2443
TMSO
OTMS
Relative intensity
m/z

Figure SM-5.

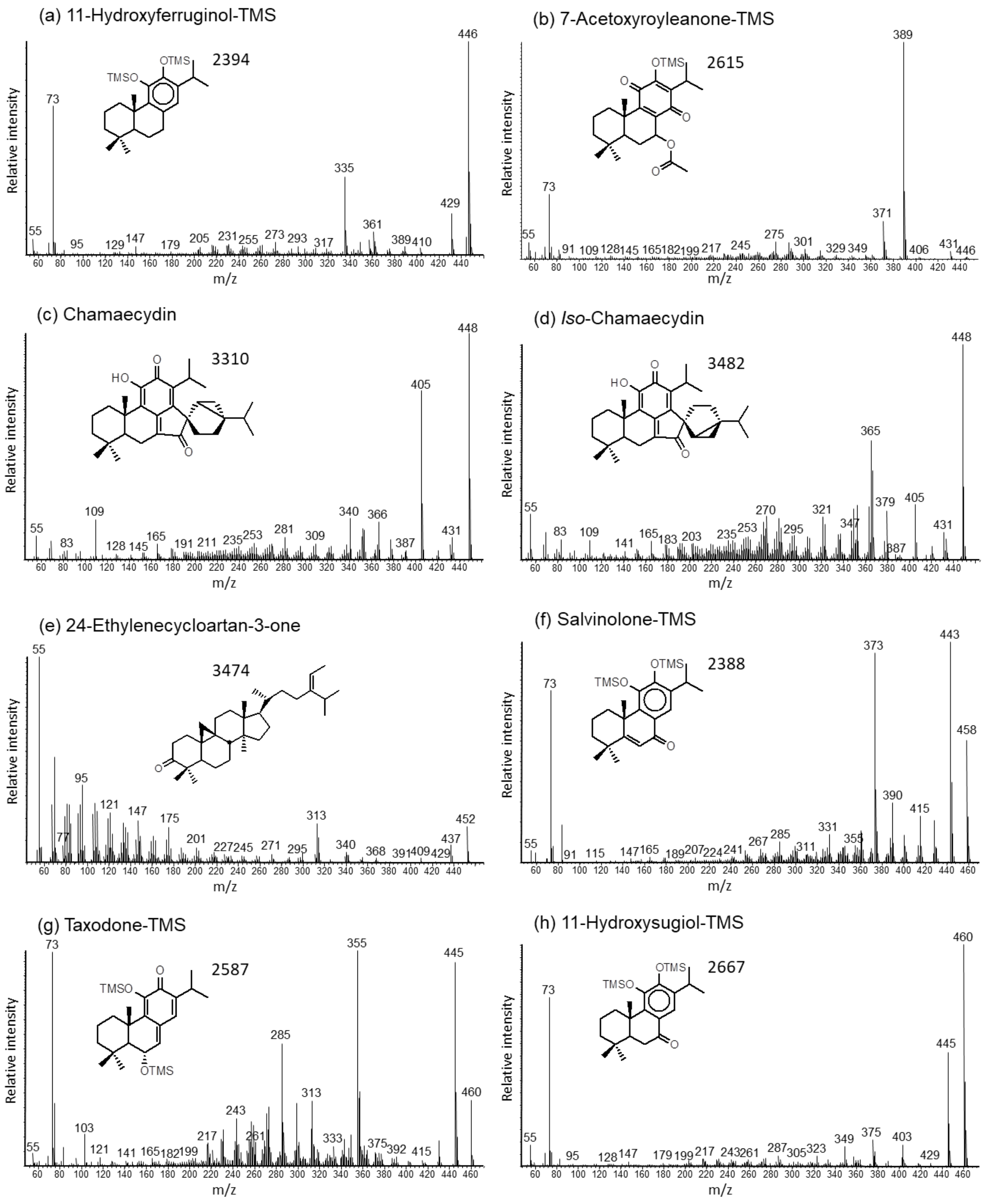
(a) 11-Hydroxyferruginol-TMS
2394
(b) 7-Acetoxyroyleanone-TMS
2615
(c) Chamaecydin
3310
(d) Iso-Chamaecydin
3482
(e) 24-Ethylenecycloartan-3-one
3474
(f) Salvinolone-TMS
2388
(g) Taxodone-TMS
2587
(h) 11-Hydroxysugiol-TMS
2667
Relative intensity
m/z

Figure SM-6.

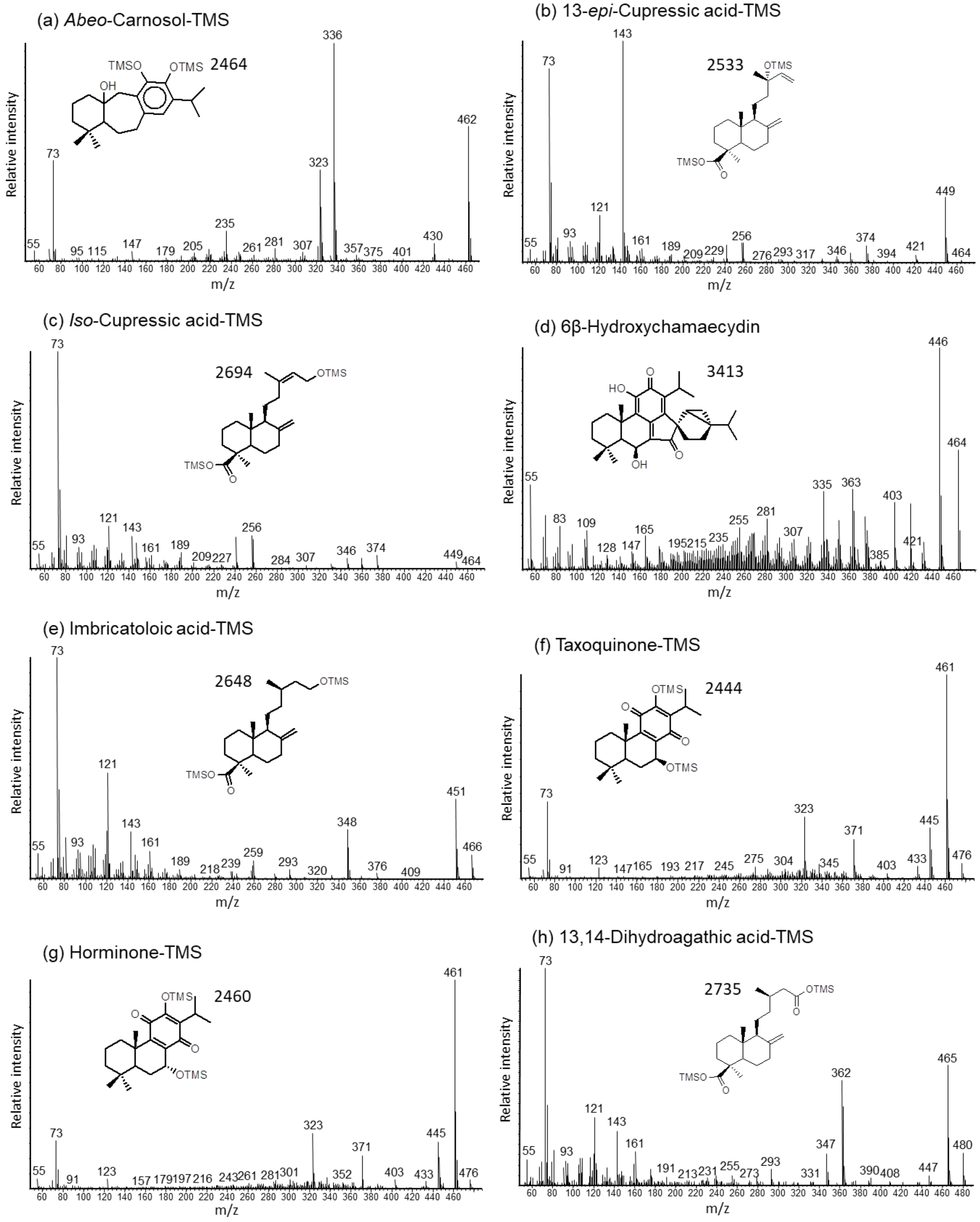
(a) Abeo-Carnosol-TMS
2464
(b) 13-epi-Cupressic acid-TMS
2533
(c) Iso-Cupressic acid-TMS
2694
(d) 6β-Hydroxychamaecydin
3413
(e) Imbricatoloic acid-TMS
2648
(f) Taxoquinone-TMS
2444
(g) Horminone-TMS
2460
(h) 13,14-Dihydroagathic acid-TMS
2735
Relative intensity
m/z

Figure SM-7.

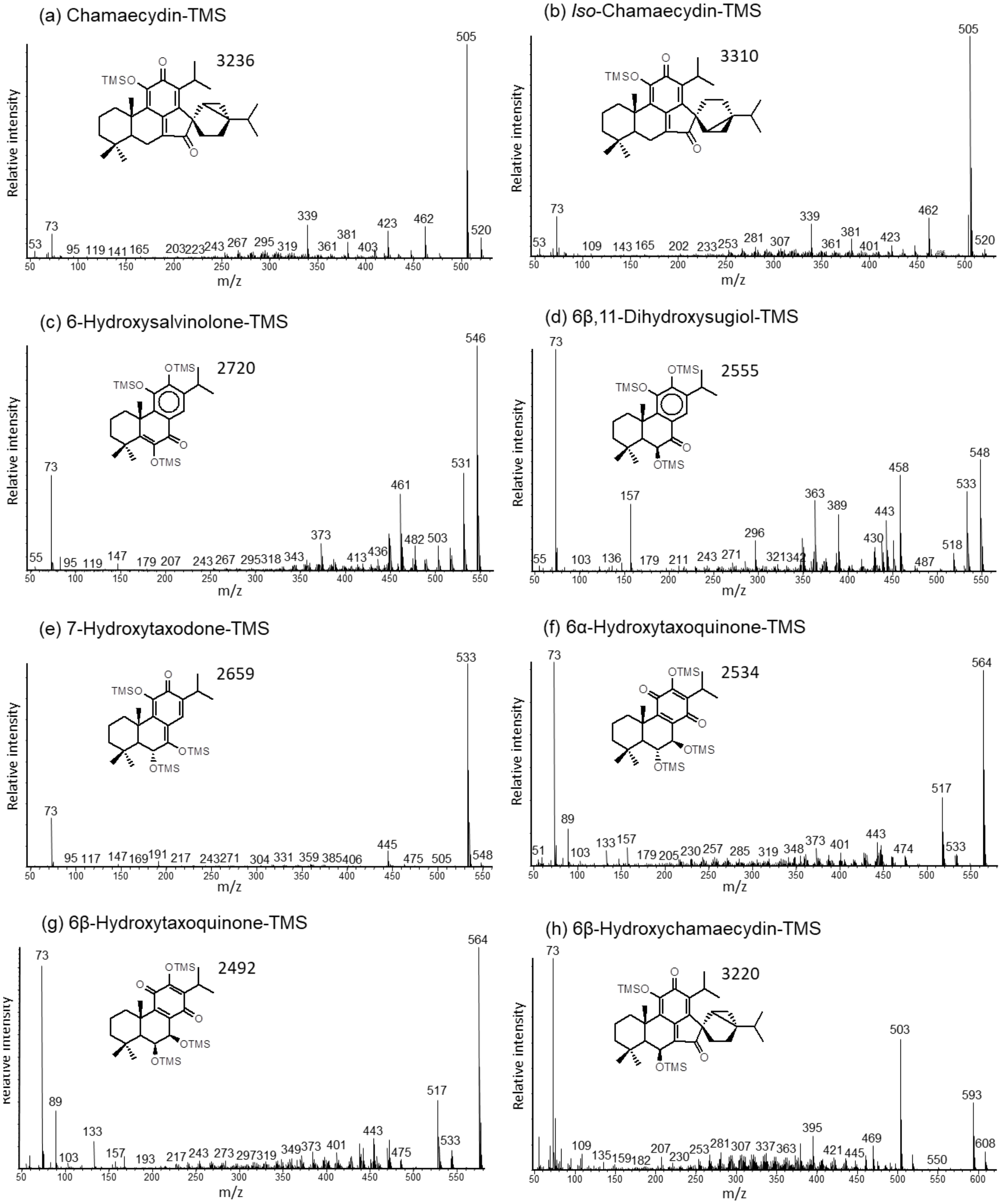
(a) Chamaecydin-TMS
3236
(b) *Iso*-Chamaecydin-TMS
3310
(c) 6-Hydroxysalvinolone-TMS
2720
(d) 6β,11-Dihydroxysugiol-TMS
2555
(e) 7-Hydroxytaxodone-TMS
2659
(f) 6α-Hydroxytaxoquinone-TMS
2534
(g) 6β-Hydroxytaxoquinone-TMS
2492
(h) 6β-Hydroxychamaecydin-TMS
3220
Relative intensity
m/z
